# Antenatally diagnosed kidney tumor: Answers

**DOI:** 10.1007/s00467-020-04857-0

**Published:** 2020-12-09

**Authors:** Wiebke Solass, Hyunkyu Shin, Cristian Urla, Andreas Schmidt

**Affiliations:** 1 Institute of Pathology and Neuropathology, University Hospital Tuebingen, Eberhard-Karls University Tuebingen, Liebermeisterstraße 8, Tuebingen, 72076 Germany; 2 Department of Pediatric Surgery and Pediatric Urology, University Children’s Hospital Tuebingen, Eberhard-Karls University Tuebingen, Hoppe-Seyler-Str. 3, 72076 Tuebingen, Germany

**Keywords:** Renal tumor, Prenatal diagnosis, Pediatric neoplasm, Nephrectomy, Congenital mesoblastic nephroma

## Answers

The tumor developed during pregnancy in the upper pole of the left kidney and displayed spindle cell components with no atypia and islands of metaplastic cartilage, which is typical for a congenital mesoblastic nephroma (CMN), classic variant. Wilms tumor (WT)/nephroblastoma is the most frequent kidney tumor in childhood, and this diagnosis has to be considered [1]. Additional immunohistochemical staining is helpful to distinguish CMN from WT with heterologous differentiation [1, 2].

## Discussion

Congenital mesoblastic nephroma represents 3% of all pediatric kidney tumors [3]. It is the most common kidney neoplasm diagnosed in the first 3 months of life, and it is frequently detected antenatally, as described in our case [4]. The malignant potential of the tumor is low.

### Histopathology

The histological classification of CMN includes three subtypes: classic, cellular, and mixed type [5]. Classic CMN is composed of braiding bundles of spindle cells and frequent metaplastic cartilage with no capsular boundaries. The tumor often infiltrates the surrounding perirenal fat tissue and parenchyma [6]. Cellular CMN also presents bundles of spindle cells but has a stronger hemangiopericytic pattern and a higher mitotic activity than the classic type. In contrast, the cellular type less frequently infiltrates the perirenal fat and/or kidney parenchyma. Mixed CMN shows, as the name indicates, a mixture of both abovementioned types.

### Molecular aspects

There are recurrent genetic aberrations described in CMN, including somatic trisomy 11 and, occurring in the cellular and mixed type, the translocation t(12;15)(p13;q25), which results in fusion of *ETV6* and *NTRK3* [7, 8]. Associations with genetic syndromes are only described in rare cases [8, 9].

## Therapy

Complete nephrectomy is curative for most patients with stage I/II disease. The removal of perirenal fat during surgery is important, as CMN tumors often show infiltrative growth into the perirenal fat. However, in case of a high risk of operative or anesthetic complications, preoperative chemotherapy may be considered. Stage III tumors of the classic and mixed histologic subtype are also indicated for nephrectomy alone. Stage III tumors of the cellular type treated only surgically have a higher rate of relapse than the other histologic subtypes requiring chemotherapy or radiotherapy in some cases. However, due to limited data, there are no specific recommendations for adjuvant chemotherapy [8]. The known side effects of radiotherapy particularly in these very young patients limit this treatment modality to selected cases with aggressive tumors not responding to chemotherapy [8].

For the targeted therapy of tumors with the *ETV6-NTRK3* fusion, tropomyosin receptor kinase inhibitors have been developed. One drug was recently approved for children with CMN and proven *ETV6-NTRK3* fusion who lack other treatment options [10]. However, conclusive data regarding the outcome of patients after application of the drug are not available.

Taking into account these aspects, it may be concluded that the decision for the adequate treatment of patients with CMN may sometimes be challenging. Therefore, the importance of an adequate diagnostic and therapeutic plan established by an interdisciplinary reference board which should include a pediatric oncologist, pediatric radiologist, pediatric surgeon, and pathologist cannot be overemphasized.

## Conclusion

In summary, CMN is a rare and in most cases a non-aggressive tumor. It is classified into three histological subtypes. Frequently, the tumor is diagnosed antenatally. Most tumors are treated with surgery only. Pre- and postoperative chemotherapy is only administered in particular cases. Nephroblastoma is a possible differential diagnosis.
